# A Modified Sliding-Lengthening Approach to Tendon Lengthening with a Locking Mechanism Suture: A Technical Note

**DOI:** 10.1007/s43465-023-00829-2

**Published:** 2023-01-30

**Authors:** Dai Iwase, Yukie Metoki, Jun Aikawa, Kentaro Uchida, Kensuke Fukushima, Takashi Matsuo, Atsushi Matsuo, Gen Inoue, Masashi Takaso

**Affiliations:** 1 Department of Orthopaedic Surgery, Kitasato University School of Medicine, 1-15-1 Kitasato, Minami-ku, Sagamihara, Kanagawa 252-0374 Japan; 2 Department of Orthopaedic Surgery, Hifumi Foundation Minamitama Othopaedic Hospital, 11-1 Onogi-machi, Machida, Tokyo 195-0064 Japan; 3 Department of Orthopaedic Surgery, Saga Handicapped Children’s Hospital, 2215-27 Kinryu, Kinryu-machi, Saga, 849-0906 Japan

**Keywords:** Tendon lengthening, Z-lengthening, Sliding-lengthening, Cerebral palsy, Locking mechanism

## Abstract

There are various techniques used for tendon lengthening, of which Z-lengthening and sliding-lengthening are the most frequently performed. In patients with cerebral palsy, tendon lengthening may often be necessary at multiple sites. However, they can cause various complications, such as inaccurate extension, overextension, and a lack of tendon continuity. We modified the sliding-lengthening technique with a locking mechanism to address these issues. This technical note aims to describe the surgical technique and pitfalls associated with the modified sliding-lengthening approach and suture locking mechanism. The tendon was exposed and stabilized using sterilized spitz tubes and was then threaded so that each loop length was equivalent to the amount of tendon extension. Symmetrical hemisection of both ends of the tendon was performed, and the tendon was carefully extended to create a tense loop. The modified sliding-lengthening technique with the locking suture mechanism may be an advantageous method that accurately addresses extension volume, prevents hyperextension, and maintains tendon continuity, even when smaller incisions are used.

## Introduction

Tendon lengthening is a useful surgical method for patients with a limited range of motion and contracture associated with muscle spasticity and has been widely reported to be used for the Achilles tendon. Although there are many different lengthening procedures, the most common are the Z-lengthening (ZL) and sliding-lengthening (SL) techniques [1]. The ZL technique has a low recurrence rate for patients suffering from equinus, but it carries a high risk of heel deformation [2]. In contrast, the SL technique is useful in decreasing the occurrence of adhesions and heel deformation. However, compared to the ZL technique, it carries a slightly higher risk of recurrence and inaccurate extension volume [3, 4]. In addition to the Achilles tendon, patients with cerebral palsy (CP) have strains in many other muscles, and treatment for each affected muscle is necessary. We improved the conventional SL technique to achieve a locking mechanism, which we believe will allow for accurate extension volume and will prevent hyperextension. In addition, the SL technique with the locking suture mechanism (SL-L) worked well for tendons other than the Achilles tendon and could be performed with a small skin incision. [5]

In this article, we present the surgical procedure and the pitfalls associated with the SL-L technique.

## Technique

**Fig. 1:**
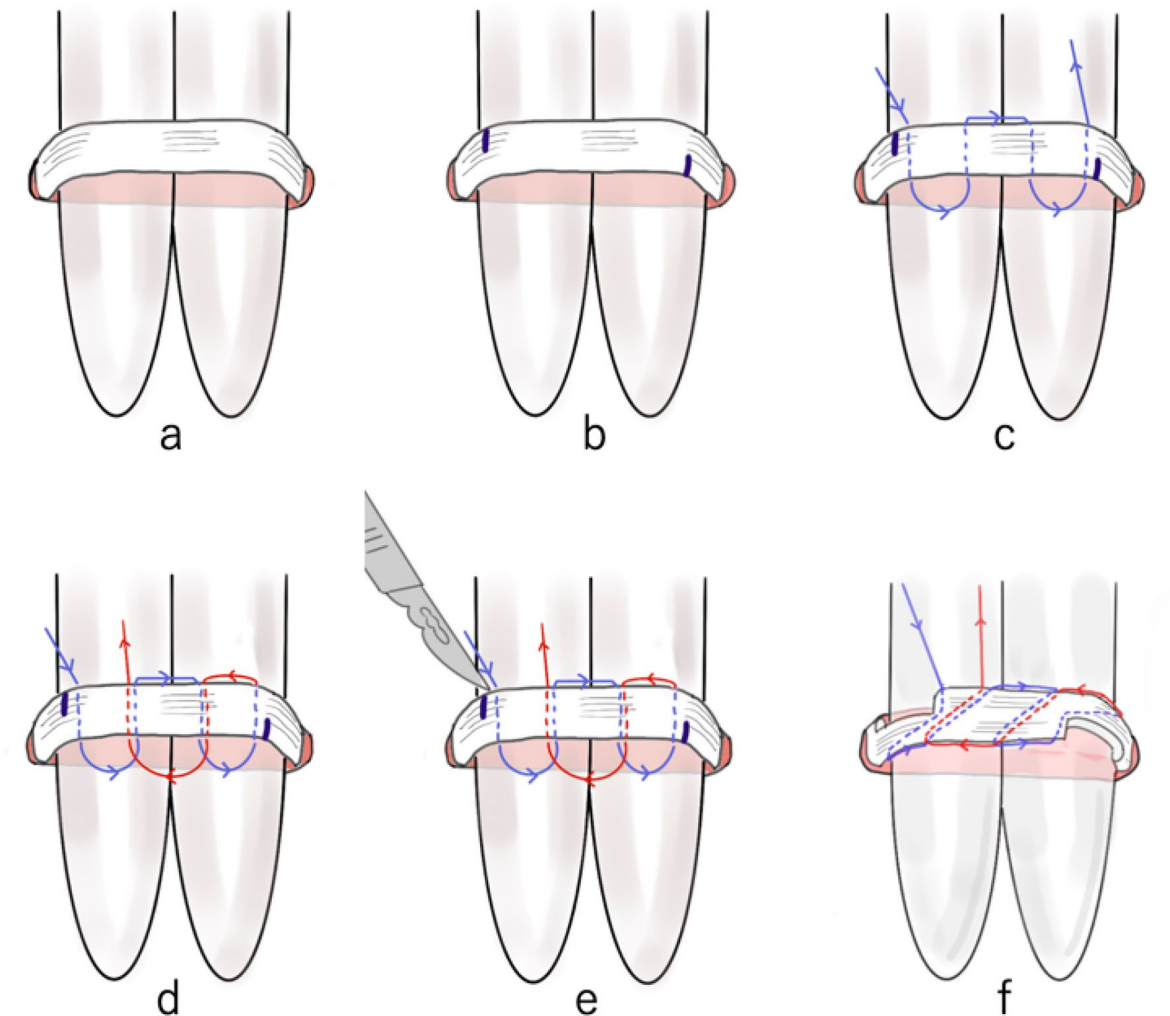
The suture procedure for tendon lengthening using the sliding-lengthening with a locking mechanism. **a** The tendon is exposed and stabilized using sterilized spitz tubes. **b** The markings show the total length of the extension and overlap. **c**, **d** Threading of the tendon. The arrows indicate the direction of threading. **e** Cutting of the tendon. The two markings indicate where the symmetrical transverse incisions are made, which are up to half the tendon width. **f** Extension of the tendon to create a tense loop

**Fig. 2:**
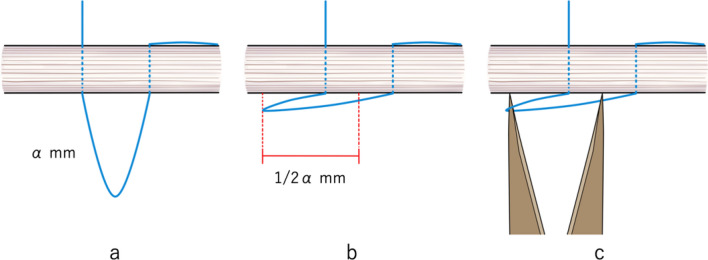
Accurate loop length measurement. **a**–**c** The loop is positioned parallel to the tendon. The distance between the apex of the loop and the center of the tendon of each loop should be half the amount of extension (1/2 *α* mm). The distance is adjusted using a Vernier caliper

An incision less than half the length of the extension was made. However, if the expanding tendon is deep (such as in psoas major muscle, or iliacus muscle), the incision may be made wider than usual. After identifying the tendon, it was exposed and stabilized using sterilized spitz tubes (Fig. 1a). Correcting the twist of the tendon was an important part of this process. Both ends of the tendon were marked depending on the length of the extension and overlap (Fig. 1b). For example, an *α* mm extension with a *β* mm overlap would require markings with a length of *α* + *β* mm. The tendon was threaded, as shown in Fig. 1c, d. At the time, the length of each loop had to be extended to the amount of tendon extension. It is particularly important to make the second loop length accurate as the locking mechanism mainly functions in the second loop. To accurately measure the loop length (α mm), the loop was made parallel to the tendon. The distance between the apex of the loop and the center of the tendon of each loop was adjusted using a Vernier caliper so that the distance was approximately half the amount of extension (1/2 *α* mm) (Fig. 2a–c). Symmetrical transverse incisions were made up to half the tendon width according to the two markings (Fig. 1e). Finally, the tendon was carefully extended to create a tense loop. After extending the tendon, the locking mechanism provided accurate volume extension and prevented overextension (Fig. 1f).

Complications associated with this technique include cut-out of the thread and rupture of the tendon at the half-section. The following pitfalls are important: (1) correcting the torsion of an exposed tendon; (2) making the second loop length more precise, as it is primarily a locking mechanism; and (3) carefully extending the tendon so that it does not break during tendon extension.

### Case presentation

**Fig. 3:**
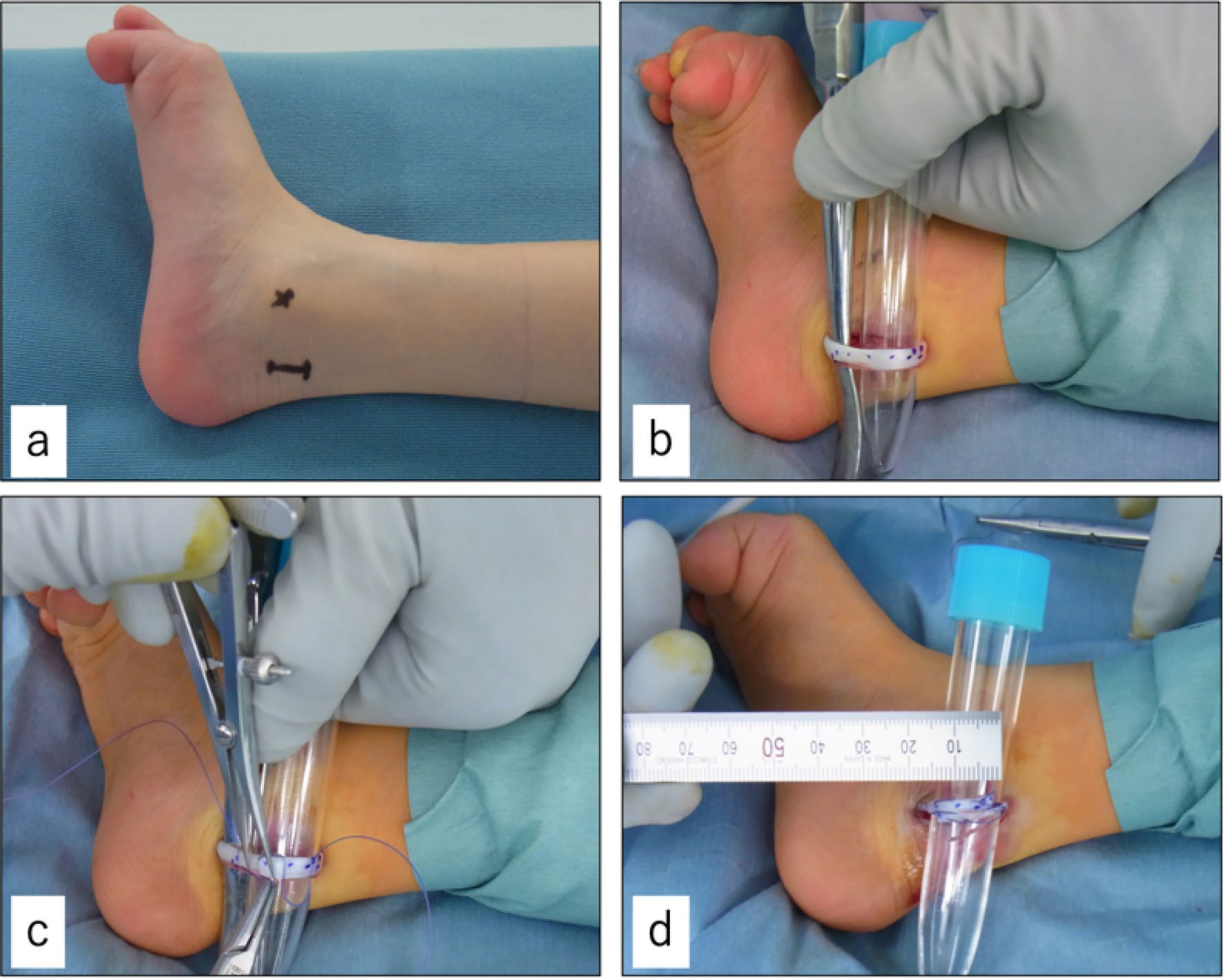
A 6-year-old boy with cerebral palsy with right lower limb monoplegia. **a** Only 15 mm skin incision was made posterior to the medial condyle of the ankle joint. **b** Flexor hallucis longus was pulled out. **c** Loop length measurement using a Vernier caliper. **d** Immediately after lengthening

The patient was a 6-year-old boy with CP, right lower limb monoplegia, and gross motor function classification system (GMFCS) level 1. Owing to the high tension of the flexor hallucis longus (FHL), we performed FHL-only lengthening. The preoperative extension angle of the big toe was -45 degrees at the metatarsophalangeal (MTP) joint and -30 degrees at the interphalangeal (IP) joint in passive extension. Only a 15 mm skin incision was made posterior to the medial malleolus of the ankle joint, as shown in Fig. 3a. We planned to perform a 15 mm extension and 15 mm overlap, and therefore, we pulled out 30 mm of the FHL and marked it (Fig. 3b). Loop length was determined to be 15 mm using a Vernier caliper (Fig. 3c). We then performed tendon lengthening and confirmed that the overlap was 15 mm (Fig. 3d). The postoperative passive extension angle of the big toe was up to 30 degrees at the MTP joint and 10 degrees at the IP joint.

## Discussion

We previously performed the SL-L technique on different tendons (finger flexor tendon [6], foot flexor hallucis tendon [7], psoas major tendon [8], and hamstring tendon) and achieved positive results. We present the clinical results of patients with equinus foot associated with ambulatory CP (Table 1). The study period was from April 2013 to June 2019, and patients who could be followed up for at least 3 years after surgery were included. Gait, GMFCS level, paralytic type, range of motion, and heel-floor distance (HFD) were used to determine the amount of extension. The amount of extension of the Achilles tendon was based on 0.4 times the HFD; for patients with HFD less than 20 mm, the fractional lengthening technique was used. If toe deformity was present, the amount of FHL extension was limited to 10–15 mm depending on the strength of the tension, while the flexor digitorum longus was limited to 15–25 mm. Tibialis posterior was added according to the degree of medial deformity, up to 20 mm. [5, 7] We observed a few cases with residual HFD postoperatively, but none of the cases with heel foot deformity had a decreased GMFCS level.

**Table 1:** Treatment results of the sliding-lengthening approach for patients with cerebral palsy with equinus foot

Case	Age (year)	BMI	Paralytic type	GMFCS	Sliding lengthening (mm)				Preoperative^a^			Postoperative^a^		
					AT	FHL	FDL	TP	DKE	DKF	HFD	DKE	DKF	HFD
1	12	18.8	Hemi	1	24	0	10	0	− 45	− 40	60	0	0	0
2	9	13.5	Hemi	2	20	8	15	5	− 30	− 30	50	10	20	0
3	20	17.5	Mono	1	30	15	25	0	− 60	− 60	70	− 7	0	0
4	6	17.7	Di	1	26	14	25	0	− 50	− 40	65	− 5	− 5	2
5	8	14.9	Mono	1	23	15	25	25	− 15	− 15	56	10	15	0
6	6	17.6	Di	1	25	13	25	0	− 50	− 40	63	− 5	− 5	2
7	5	15.6	Hemi	1	30	12	18	0	− 60	40	70	10	20	0
8	20	22.6	Mono	1	18	13	25	15	− 40	− 40	40	− 5	0	0
9	30	23.8	Para	3	20	12	35	20	− 60	− 55	50	0	10	0
10	30	24.1	Para	3	18	10	30	15	− 50	− 45	40	0	0	5
11	6	16.2	Hemi	1	18	10	12	0	− 50	− 20	45	− 5	5	5
12	8	17.4	Mono	1	25	10	15	0	− 25	− 25	60	5	10	5
13	9	16.9	Di	3	35	15	12	15	− 50	− 30	95	5	0	10
14	12	26.2	Para	1	40	15	20	0	− 45	− 40	85	0	5	0
15	52	20.2	Mono	3	22	9	12	0	− 20	− 20	50	0	10	0
16	10	16	Quad	3	20	10	15	12	− 15	0	50	5	10	0
17	8	14.2	Hemi	2	10	8	0	10	− 50	− 40	20	5	10	0
18	12	18.8	Di	1	20	0	10	0	− 45	− 40	60	0	0	0
19	9	13.5	Hemi	2	20	8	15	5	− 30	− 30	50	10	20	0
20	20	17.5	Mono	1	35	15	25	0	− 60	− 60	70	− 7	0	0

*BMI* body mass index, *GMFCS* gross motor function classification system, *AT* Achilles tendon, *FHL* flexor hallucis longus, *FDL* flexor digitorum longus, *TP* tibialis posterior, *DKE* dorsiflexion with knee extension, *DKF* dorsiflexion with knee flexion, *HFD* heel-floor distance, *Hemi* hemiplegia, *Mono* monoplegia, *Di* diplegia, *Para* paraplegia, *Quad* quadriplegia

^a^DKE and DKF are in degrees, FHD is in mm

The features that make this technique advantageous are the accuracy of the amount of extension as well as the prevention of overextension due to the locking suture mechanism before performing an extension. Thus, the SL-L technique has improved the maintenance of collagen fiber continuity compared to the conventional SL or ZL technique and is considered stronger immediately after extension. In addition, in an in vivo rabbit model, the SL-L technique had improved mechanical properties up to 3 weeks postoperatively compared to the ZL technique [9]. Thus, it may allow for a shorter postoperative immobilization period. For example, the casting plan after Achilles tendon lengthening was above-knee cast immobilization for 3 weeks postoperatively and below-knee cast immobilization for the following 3 weeks; however, we believe that the SL-L technique may reduce the postoperative casting period or change the above-knee casting period to below-knee casting. Moreover, we believe that this method makes it easier to achieve tendon lengthening in sites other than the Achilles tendon as it allows the tendon to be pulled out and sutured with a small incision.

## Conclusion

The SL-L technique can be performed with a small incision, and the locking mechanism prevents overextension of the tendon, allowing for an adequate amount of lengthening. Thus, this technique addresses the complications faced during tendon lengthening, such as extension volume, to prevent hyperextension and maintain tendon continuity.

## Data Availability

The datasets supporting the conclusions of this article are included within the article. The corresponding author can provide raw data on request.
